# Linear and nonlinear optical properties of transfer ribonucleic acid (tRNA) thin solid films

**DOI:** 10.1039/d1ra09412b

**Published:** 2022-03-18

**Authors:** Marjan Ghasemi, Hayoung Jeong, Donggyu Kim, Byungjoo Kim, Joon Ik Jang, Kyunghwan Oh

**Affiliations:** Department of Physics, Photonic Device Physics Laboratory, Yonsei University 50 Yonsei-ro Seodaemun-gu Seoul 120-749 South Korea koh@yonsei.ac.kr; Center for Quantum Information, Korea Institute of Science and Technology (KIST) Seoul 02792 South Korea; Department of Physics, Nonlinear Optical Material & Spectroscopy, Sogang University 35 Baek-beom-ro Seoul 04107 South Korea

## Abstract

We successfully obtained transfer ribonucleic acid (tRNA) thin solid films (TSFs) using an aqueous solution precursor in an optimized deposition process. By varying the concentration of RNA and deposition process parameters, uniform solid layers of solid RNA with a thickness of 30 to 46 nm were fabricated consistently. Linear absorptions of RNA TSFs on quartz substrates were experimentally investigated in a wide spectral range covering UV–VIS–NIR to find high transparency for *λ* > 350 nm. We analyzed the linear refractive indices, *n*(*λ*) of tRNA TSFs on silicon substrates by using an ellipsometer in the 400 to 900 nm spectral range to find a linear correlation with the tRNA concentration in the aqueous solution. The thermo-optic coefficient (d*n*/d*T*) of the films was also measured to be in a range −4.21 × 10^−4^ to −5.81 × 10^−4^ °C^−1^ at 40 to 90 °C. We furthermore characterized nonlinear refractive index and nonlinear absorption of tRNA TSFs on quartz using a Z-scan method with a femtosecond laser at *λ* = 795 nm, which showed high potential as an efficient nonlinear optical material in the IR spectral range.

## Abstract 2

Optical measurements of one of the vital biological molecules (RNA) in the human body.

## Graphical abstract

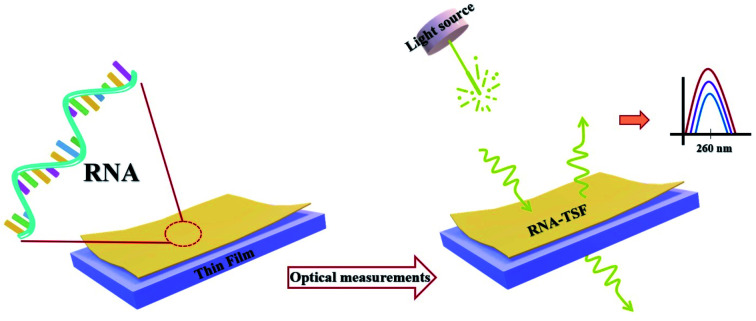

## 1. Introduction

Deoxyribose nucleic acid (DNA) has recently shown prominent potential as a highly functional electro-optic material.^1,2^ Thin solid films (TSFs) made from either DNA aqueous solutions^3^ or other organic solutions^4–7^ have found unique optoelectronic applications. For instance, DNA-TSF has been used as an electron blocking layer in organic light-emitting diodes (OLEDs),^8,9^ hydrogen-blocking layer in hybrid thin-film transistors (TFTs),^10,11^ a cladding layer in an optical waveguide modulator,^12^ a sensing medium in optical sensors,^13,14^ a nonlinear saturable absorber in femtosecond lasers,^3^ and a radiation absorbing layer in solar cells,^15,16^ to name a few. In contrast to DNA, ribonucleic acid (RNA) research has been primarily confined to traditional biochemical and pharmaceutical usages.^17–19^ In recent years, RNA-based vaccines have become a preventive necessity to curb the global pandemic.^20–28^ While DNA is a double strand, RNA forms a single strand of the nucleobase arrays and is categorized into messenger RNA (mRNA), transfer RNA (tRNA), and ribosomal RNA (rRNA).^20^ RNA shares its constituents, adenine (A), guanine (G), cytosine (C), with DNA. Uracil (U) in RNA replaces thymine (T) in DNA.

RNA compounds are more resistant against UV radiation than DNA counterparts,^25,29^ which makes RNA TSF advantageous in an environment exposed to radiation containing UV spectral components. In RNA aqueous solution, we experimentally found that its viscosity was significantly lower than DNA solutions for the same concentration level. RNA aqueous solution precursors provided a superb thin film fabrication capability, especially in the spin coating process, compared to DNA precursors. Despite nearly the same constituents as DNA, RNA has not been thoroughly studied as an optoelectronic substance, and especially RNA TSFs for photonic applications have not been reported to the best knowledge of the authors.

In this study, we report a systematic process to fabricate tRNA TSFs with various thicknesses on both silicon (Si) and silica substrates using a surfactant-free aqueous solution of tRNA from wheat germ,^6,10^ for the first time. We further analyzed their linear optical properties in terms of absorption, *α*, in the UV–VIS–NIR spectral range, linear refractive index, *n*(*λ*), in the visible to NIR from 400 to 900 nm, and thermo-optic coefficient, d*n*/d*T*, in the temperature range from 40 to 90 °C. Utilizing these linear optical properties, we furthermore investigated the nonlinear refractive index, *n*_2_, and the nonlinear absorption, *β*, using a femtosecond laser Z-scan technique,^30^ revealing the high potential of tRNA TSF in nonlinear optical applications.

**Fig. 1:**
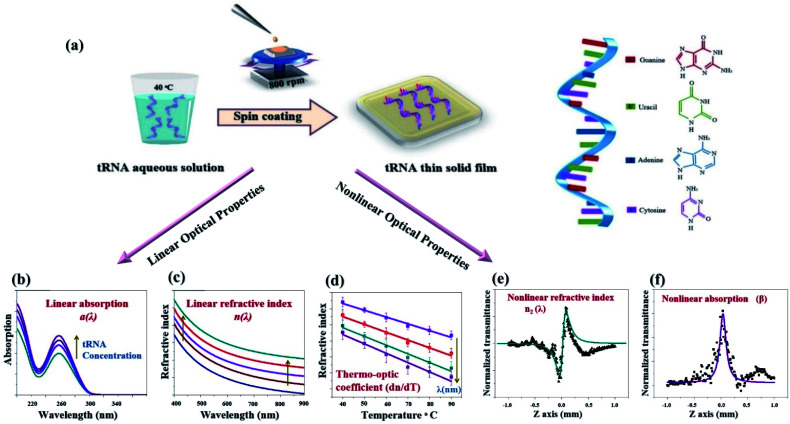
The schematic diagram for our experimental studies to fabricate tRNA TSFs and quantify their optical properties in both linear and nonlinear regimes. (a) Dissolving tRNA powder in DI water to prepare the solution precursor, and deposition of tRNA TSFs by optimizing the spin coating process. (b)–(d) Controlling the linear optic properties, *n*(*λ*), *α*(*λ*), and d*n*/d*T* of tRNA TSFs by varying tRNA concentrations. (e and f) Nonlinear refractive index *n*_2_ and nonlinear absorption *β* of tRNA film using Z-scan technique.

A schematic diagram for our study is presented in (Fig. 1). We developed a process to prepare a tRNA aqueous solution with a flexibly controllable tRNA concentration. To cope with the low viscosity of the tRNA solution precursor, we optimized the spin coating and drying process to obtain optical quality TSFs.

For these samples, we experimentally investigated the linear optical properties (*n*, *α*, and d*n*/d*T*) and nonlinear optical characteristics (*n*_2_ and *β*). It was found that the linear optical characteristics were dependent upon the concentration of tRNA prior to thin film deposition. Experimental details are discussed in the following sections.

## 2. tRNA thin-film deposition

**Fig. 2:**
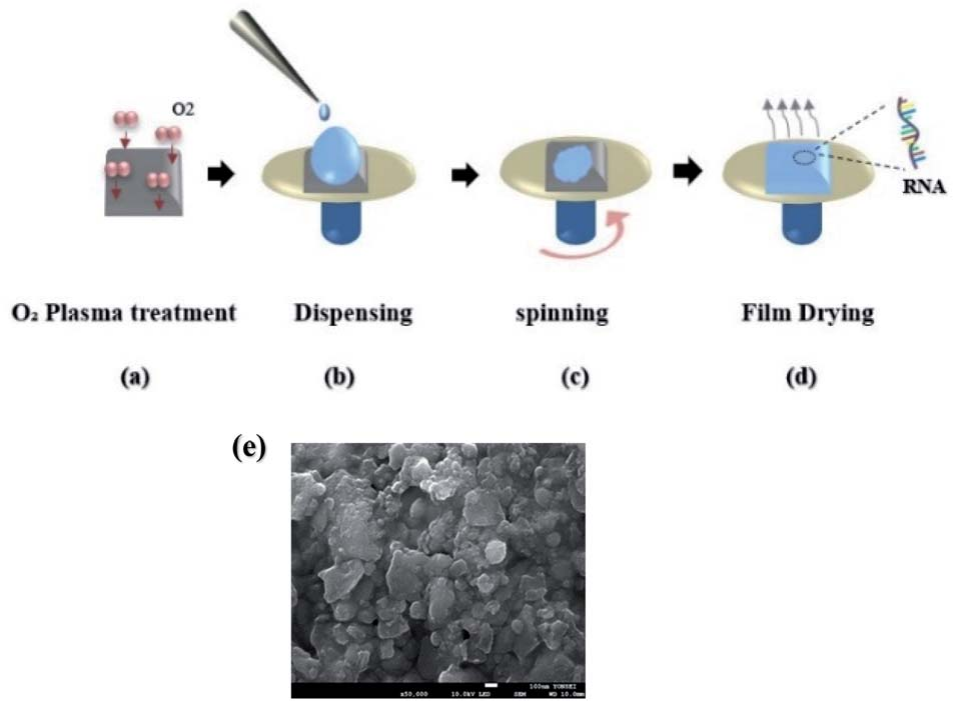
(a) Oxygen plasma treatment on quartz/silicon substrates. (b) Dispensing tRNA solution on the treated substrate. (c) Spinning the dispensed aqueous precursor on the substrate. (d) Drying the deposited layer to make tRNA thin solid film on the substrate. (e) SEM image of a prepared tRNA thin solid film.

We used commercially available tRNA powder processed from wheat germs (Sigma-Aldrich) with high purity.^31,32^ In contrast to DNA solutions used in reports 3, 5, 6, we found that tRNA required an additional process to be fully dissolved in deionized water. In a vial filled with DI water, we added the tRNA powder, and then the solution was heated at 40 °C for several hours until no precipitations were observed. To prepare a high concentration DNA solution, the solution had to be stirred for ∼24 hours^3,4,6,7^ at room temperature preventing polymerization. On the while, RNA solution was prepared within two hours with an additional heating process, and we did not observe any sign of polymerization. Note that we did not use either surfactants or other chemicals to prepare the solution. In the wholly dissolved aqueous solutions, tRNA concentration varied from 0.06 to 2.0 wt%. We used the spin coating process to deposit the tRNA layer on substrates for the prepared aqueous precursor, as depicted in (Fig. 2a–d).

We prepared the quartz and silicon substrates in the following steps: ultrasonic cleansing in acetone and isopropyl alcohol for 5 minutes each, nitrogen (N_2_) drying, oxygen plasma surface treatment. See (Fig. 2a). These steps improved the surface hydrophilicity to uniformly wet the substrates with the aqueous solution, as shown in (Fig. 2b). The dispensed tRNA solution volume ranged from 0.03 to 0.1 mL. A commercial spin-coater (ACE-200) was used at a rotation rate of 800 rpm for 6 minutes to form a thin wet film on the substrate, as shown in (Fig. 2c). In (Fig. 2d), tRNA films were vacuum dried in a desiccator at 20 °C for 24 hours to reduce the remnant moisture inside the film^33^ (Fig. 2e). Exhibits a scanning electron microscopy (SEM) image of spin-coated tRNA on a quartz substrate, confirming a homogeneous surface morphology without significantly large grain boundaries. The film thickness was in the range of 30–46 nm, controlled by the tRNA concentration in the aqueous solution.

## 3. Results and discussion

### 3.1 Absorption spectra of tRNA aqueous solution precursor

**Fig. 3:**
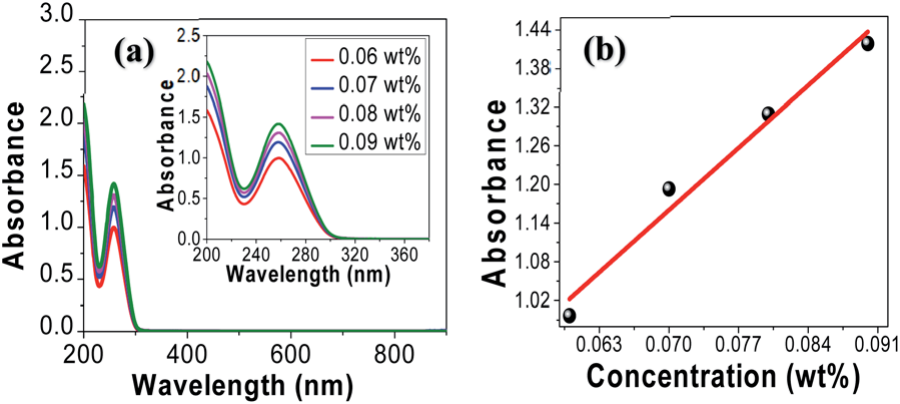
(a) Absorption spectra of tRNA aqueous solutions. (b) Peak absorbance near *λ* = 260 nm *versus* tRNA concentration.

Before investigating tRNA TSF, we began with tRNA aqueous solutions precursor to characterize their linear absorptions in UV–visible–near IR range. We prepared tRNA aqueous solutions with various concentrations from 0.06 to 0.09 wt%. We poured the solution into a 1 mm path-length quartz cuvette to measure its absorbance, using a commercial spectrometer (V-650, JASCO Corporation) in the spectra range of 200–900 nm. The results are summarized in (Fig. 3a). The tRNA solutions showed an absorbance peak around *λ* = 260 nm which was attributed to the π–π* transition of the nucleic bases.^34–39^ In (Fig. 3b), the peak absorbance linearly increased with tRNA concentration.^40^

### 3.2 Absorption spectra of tRNA thin solid films

**Fig. 4:**
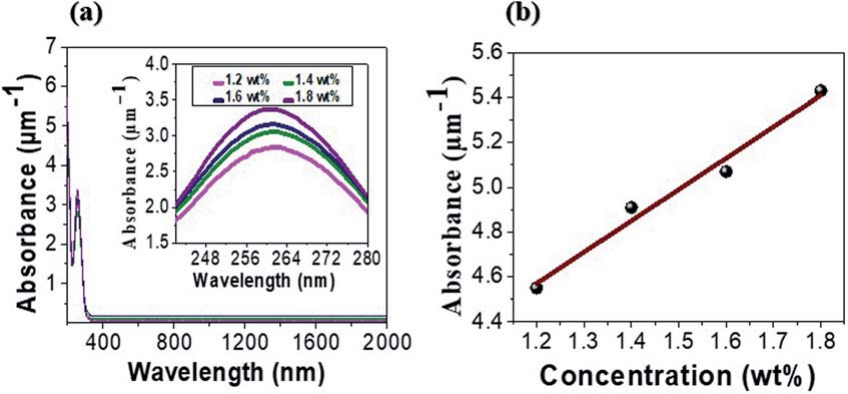
(a) Absorption spectra of tRNA thin solid films for various tRNA concentrations. (b) The peak absorbance *versus* tRNA concentration.

We fabricated tRNA thin solid films on quartz substrates by spin-coating process, using the process described in Section 2. Absorption spectra of tRNA TSFs were measured using a spectrophotometer (Cary 5000, Agilent), and the results are summarized in (Fig. 4a). The peak absorption was near *λ*_max_ ∼ 260 nm, the same as in the aqueous solution in (Fig. 3a). The similarity of the absorption spectra (Fig. 3a), and (Fig. 4a) strongly indicates that tRNAs were successfully embedded in thin solid films without significant structural modifications. In (Table 1), the average film thickness for various tRNA concentrations is plotted.

**Table 1:** The average thickness of tRNA thin solid films prepared by the spin-coating process

tRNA concentration (wt%)	Thin-film thickness (nm)
1.2	30
1.4	35
1.6	39
1.8	41
2.0	46

In (Fig. 4a), TSFs were nearly transparent for *λ* > 300 nm, showing a high potential as a transparent optoelectronic passive material.^41–43^ The peak absorbance of tRNA TSFs was more larger than four times larger than those of aqueous solutions, as shown in (Fig. 4b). This is a significant increase in the order of 10^6^ if we compare the film thickness of 30–46 nm with the cuvette path of 1 mm for the solutions. The nucleobases of RNA are composed of benzene rings, and these rings are known to form the stable π–π stacking.^44,45^ This attractive, noncovalent interaction between aromatic rings naturally increases the density of RNA in TSF and subsequently increases the absorption compared to the solution precursors. An increase of absorption in TSF compared to the solution precursors has also been observed in DNA.^4,6^ In (Table 1), we also summarized how the thin film thickness could be controlled by the tRNA concentration in the precursor solution. The film thickness was flexibly controlled in the range of 30 to 46 nm, and it monotonically increased with tRNA concentration.

### 3.3 Optical dispersion of tRNA thin films

**Fig. 5:**
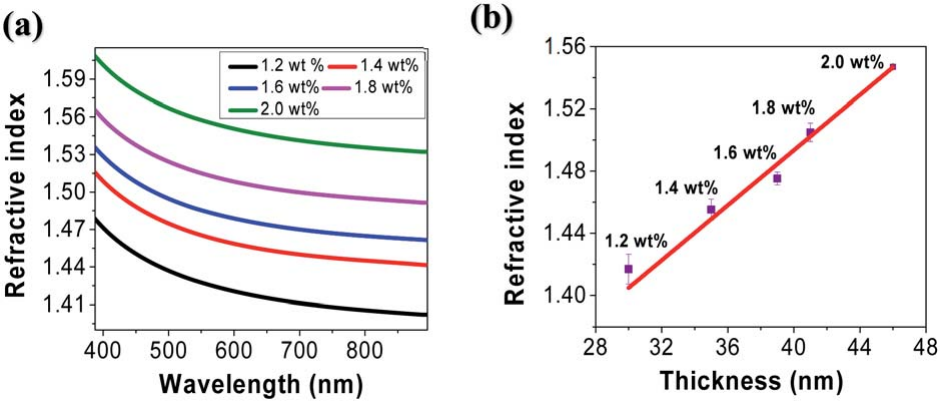
(a) The refractive index of tRNA thin solid film in the spectral range from 400 nm to 900 nm for various tRNA concentration. (b) The refractive index *versus* tRNA film thickness at *λ* = 633 nm.

Optical dispersion characteristics of tRNA TSFs were measured from 400 to 900 nm using a Wollam ellipsometry system. The results are shown in (Fig. 5a). TSF thickness was measured by applying ellipsometer.^46–48^ The film thickness was estimated by interference between light reflecting from the surface and light traveling through the film for various polarization and wavelength. In the calculation, we used the isotropic Cauchy model^2,3^ because there were no resonant absorption bands in the corresponding spectral range, as shown in (Fig. 4). The refractive index at *λ* = 633 nm increased from ∼1.42 to ∼1.56 for the increasing tRNA concentration from 1.2 to 2.0 wt%. The refractive index and the absorbance of material are correlated by Kramers–Kronig (K–K) relations.^7^*n*(*λ*) in (Fig. 5a), and *α*(*λ*) in (Fig. 4a) for the tRNA TSFs are consistently explained by K–K relation. The refractive index change in (Fig. 5a) is sufficiently large to make an all-RNA waveguide, which is being pursued by the authors. Using the film thickness data in (Table 1), we plotted the refractive index at *λ* = 633 nm *versus* film thickness in (Fig. 5b) to find a linear correlation between the film thickness and the refractive index, which was similar to DNA films.^4^ This behavior was attributed to the surface interaction to orient molecules.^49,50^ Note that we took all the measurements in a laboratory environment maintained at a temperature of ∼20 °C, and humidity of 40–50%. However, these films may be subject to gradual changes in the thickness and refractive index due to interactions with the biological substances.^7,51,52^ And the reliability issues of the optical characteristics of the tRNA TSFs are being investigated by the authors.

### 3.4 Thermo-optic coefficient of tRNA thin solid films

**Fig. 6:**
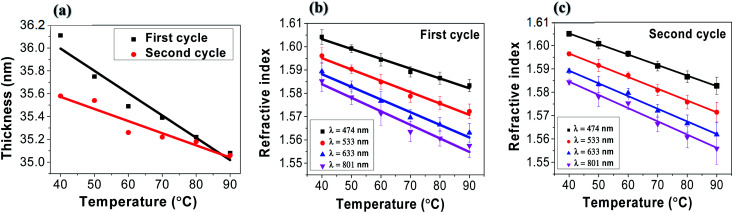
(a) tRNA thin solid film thickness as a function of temperature in two separate thermal cycles. (b) Refractive index in the 1st cycles, (c) refractive index in the 2nd cycles.

We investigated the change in the refractive index of tRNA TSFs as a function of temperature to find the thermo-optic coefficient, d*n*/d*T*. By mounting tRNA TSF on a Peltier thermoelectric device installed at an ellipsometer, we were able to measure its *n*(*λ*) in the temperature range from 40 to 90 °C, and the results are summarized in (Fig. 6).

We repeated two temperature cycles with a heating/cooling rate of ∼10 °C minute^−1^ for a tRNA TSF on Si substrate with an initial thickness of 46 nm made from 2.0 wt% tRNA solution. In experiments, the humidity was 40–50%. We noted that the film thickness decreased with the increasing temperature, as shown in (Fig. 6a). In both cycles, the tRNA TSF thickness decreased linearly with the rising temperature, and its slope decreased in the 2nd temperature cycle. This behavior is similar to DNA films in prior reports,^4,6^ which has been attributed to the swelling of films by the surrounding humidity. In (Fig. 6b and c), we plotted the refractive index *versus* temperature for 1st and 2nd temperature cycles, respectively. In both figures, tRNA TSFs showed a linear decrease in the refractive index with increasing temperature. The linear regression obtained the slope corresponding to d*n*/d*T* and compared it with DNA films in (Table 2). It is noted that d*n*/d*T* of tRNA is slightly smaller than that of DNA, which might be related to tRNA's single strand structure.

**Table 2:** Thermo-optic coefficient (×10^−4^ °C^−1^) of tRNA 2 wt% and DNA thin solid film (TSF)

*λ* (nm)	tRNA TSF		DNA TSF^4^	
	d*n*/d*T* 1st cycle	d*n*/d*T* 2nd cycle	d*n*/d*T* 1st cycle	d*n*/d*T* 2nd cycle
474	−4.21	−4.50	—	—
533	−4.86	−5.02	−5.16	−5.19
633	−5.43	−5.42	−5.81	−5.75
801	−5.81	−5.66	—	—

## 4. Nonlinear optical properties of tRNA thin solid film measured by Z-scan method

**Fig. 7:**
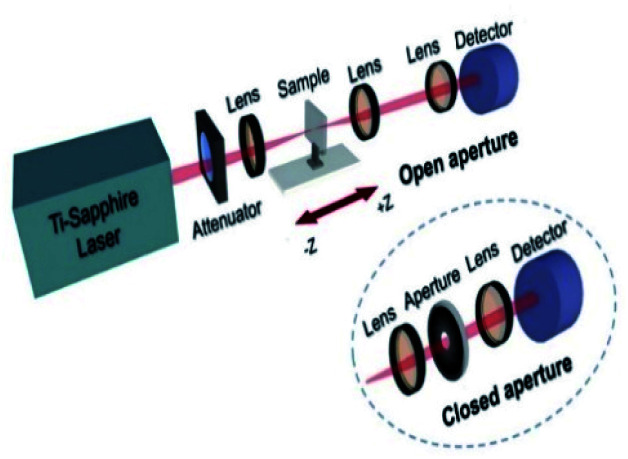
Schematic of the Z-scan setup for the open aperture and the closed aperture configuration.

We experimentally investigated the third-order nonlinear optical properties of tRNA TSFs using the Z-scan method.^30,53^ We showed the experiment schematic in (Fig. 7). We implemented both open- and closed-aperture configurations, using a Ti-Sapphire laser (Tsunami, Spectra-Physics) at *λ* = 795 nm with the repetition rate of 80 MHz and the pulse duration of 90 fs. At the focal point, the laser-beam intensity was about 17.6 GW cm^−2^ with a beam radius of 7 μm.

To endure the high laser intensity, we prepared tRNA film with a thickness of ∼8.86 μm using the drop-casting method.^54,55^

**Fig. 8:**
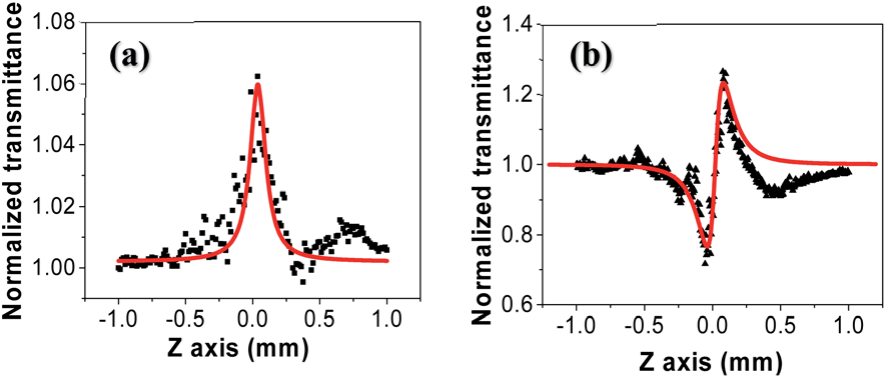
(a) Open-aperture and (b) closed-aperture Z-scan measurements of tRNA film, superimposed with best fits to the data shown in red curves.

The normalized transmittance along the *z*-axis was measured for both open and closed configurations, and the results are shown in (Fig. 8a and b), respectively. The open-aperture data was fitted by eqn (1):1
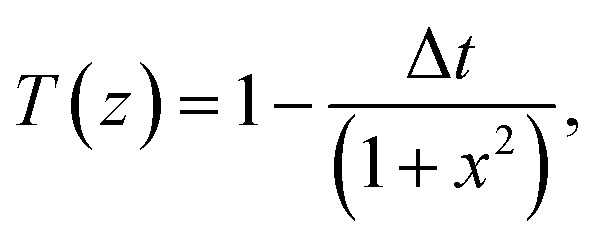
where Δ*t* is a fit parameter, and the best fit is shown in a red curve in (Fig. 8a). The red trace in (Fig. 8b) corresponds to our fit to the closed-aperture scan with eqn (2):^30^2
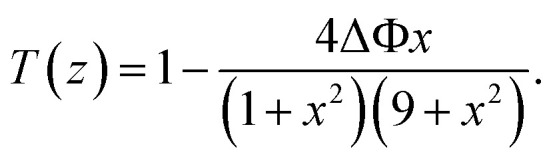


Then, the nonlinear absorption *β* was estimated by eqn (3):3
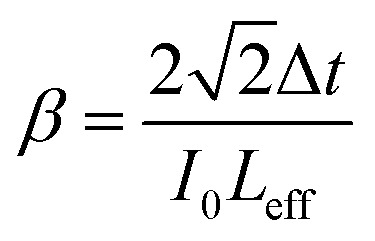
where *I*_0_ is the laser intensity at the focal point and *L*_eff_ = (1 − e^−*αL*^)/*α* is the effective thickness of the sample, calculated with the linear absorption coefficient *α* = 0.01588 μm^−1^ and the actual sample thickness *L*. The nonlinear refractive index *n*_2_ was estimated by eqn (4):4
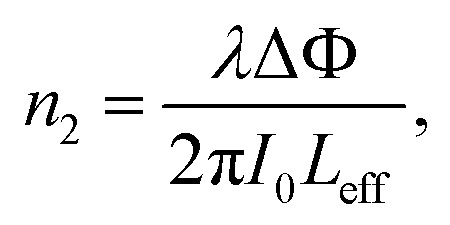
where *λ* is the laser wavelength and Δ*Φ* is the nonlinear phase change.

We measured *β* ∼ −1.12 × 10^−10^ m W^−1^ in tRNA film, where the negative sign indicates the saturable absorption capability. We also estimated *n*_2_ ∼ 1.00 × 10^−12^ cm^2^ W^−1^, where the positive sign was due to the significant difference between the laser energy and the bandgap of tRNA (>∼4.7 eV).^56^ We compared both *β* and *n*_2_ of tRNA with those of DNA and DNA-CTMA films in (Table 3). Note that *n*_2_ of tRNA is about three times larger than that of DNA. The large negative *β* and the large positive *n*_2_ of tRNA film strongly indicate that it can have a high potential as a nonlinear optical material used in mode-locking and Q-switching for short pulse generation.^57–59^ The authors are investigating pulse generation using tRNA TSF as a saturable absorber and will report the results in a separate article.

**Table 3:** The nonlinear absorption coefficient (*β*) and the nonlinear refractive index (*n*_2_) of tRNA and DNA thin solid films

Wavelength (nm)	Material	*β* (m W^−1^)	*n*_2_ (cm^2^ W^−1^)	Ref.
795	RNA	−1.12 × 10^−10^	1.00 × 10^−12^	This study
800	DNA	−1.51 × 10^−10^	3.63 × 10^−13^	3
800	DNA-CTMA	−7.71 × 10^−10^	1.81 × 10^−12^	3

## 5. Conclusion

We successfully developed a new method to fabricate surfactant-free tRNA thin solid films (TSFs) by using tRNA aqueous solution precursors and we precisely controlled their optical characteristics. Both linear and nonlinear optical properties of tRNA TSFs showed their high potential in all-RNA photonic device applications. By measuring the absorbance, we could confirm that tRNA were successfully imbedded in solid films without structural changes. The refractive index at *λ* = 633 nm increased from ∼1.42 to ∼1.56 as tRNA concentration increased from 1.2 to 2.0 wt%. The high refractive index difference of Δ*n* > 0.1 in the visible–near IR region controllable by tRNA concentrations confirmed the high potential of TSFs in biocompatible optical waveguide applications. The refractive index was also controlled systematically by temperature. We obtained thermo-optic coefficients (d*n*/d*T*) in a range from −4.21 to −5.81 × 10^−4^ °C^−1^ for 2.0 wt% tRNA TSF, which suggests further applications to temperature sensing and thermo-optic devices. Nonlinear optical properties of tRNA TSFS were characterized by the Z-scan method, and we estimated a large negative nonlinear absorption of *β* ∼ 1.12 × 10^−10^ m W^−1^ and positive nonlinear refractive index *n*_2_ ∼ 1.00 × 10^−12^ cm^2^ W^−1^ at *λ* = 795 nm, which were comparable or larger than those of DNA films. The large magnitude of *β* and *n*_2_ strongly indicates tRNA TSFs can open new nonlinear optical applications.

## Conflicts of interest

There are no conflicts to declare.

## Supplementary Material
